# Synthesis and Antibacterial Activity of Amino Acid and Dipeptide Prodrugs of IMB-070593, a Fluoroquinolone Candidate

**DOI:** 10.3390/molecules19056822

**Published:** 2014-05-23

**Authors:** Tingting Zhang, Jinwei Wu, Shihong Chen, Kaixiang Liu, Yabin Lin, Huiyuan Guo, Mingliang Liu

**Affiliations:** 1 Institute of Medicinal Biotechnology, Chinese Academy of Medical Sciences and Peking Union Medical College, Beijing 100050, China; 2 Zhejiang Starry Pharmaceutical Co. Ltd., Xianju 317300, China; 3 China Resources Double-Crane Pharmaceutical Co. Ltd., Beijing 100102, China; 4 Harbin Stomatological Hospital, Harbin 150010, China

**Keywords:** IMB-070593, prodrugs, synthesis, water solubility, antibacterial activity

## Abstract

A series of amino acid and dipeptide prodrugs of IMB-070593, a fluoroquinolone candidate discovered in our lab, were synthesized and evaluated for their water solubility and then antibacterial activity. Our results reveal that four amino acid prodrugs **4a**,**b**,**e**,**f** and two dipeptide prodrugs **4k**,**l** have much greater solubility (>85 mg/mL) than IMB-070593 mesylate (22.5 mg/mL). Compounds **4a** and **4k** show good *in vivo* efficacy against MSSA 12-1 (p.o./i.v., 5.32–7.68 mg/kg) and *S. pneumoniae*12-10 (p.o., 18.39–23.13 mg/kg) which is 1.19–1.50 fold more active than the parent drug.

## 1. Introduction

Fluoroquinolone (FQ) antibacterial agents which target two type II bacterial topoisomerase enzymes, DNA gyrase and/or topoisomerase IV, are among the most attractive drugs in the anti-infective chemotherapy field [1].

The structure activity relationship (SAR) studies of FQs show that the basic substituent at the C-7 position, the most amenable site for chemical changes, greatly influences their antibacterial potency, spectrum and safety [2]. The presence of five- or six- membered nitrogen heterocycle including pyrrolidine, piperazine and piperidine at this position is a particularly favorable structural feature of important FQs on the market [3]. Moreover, methyloxime-functionalized pyrrolidines as novel C-7 substituents have also been proved to be of importance with respect to biological activity and led to the discovery of some new FQ agents, such as gemifloxacin, zabofloxacin and DW286 [4,5,6].

**Figure 1:**
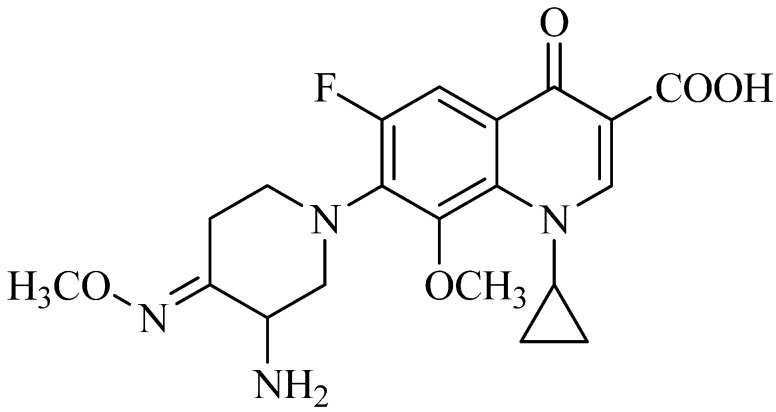
Structure of IMB-070593.

Recently, as a part of an ongoing program to optimize FQs against bacterial pathogens and mycobacterium tuberculosis (MTB), we have focused our attention on exploring the effect of introducing an oxime group into azetidine, pyrrolidine or piperidine side chains at the C-7 position of FQs, and some of them were found to have considerable biological activity [7,8,9,10]. For example, IMB-070593 (Figure 1), a piperidinyl-based FQ candidate discovered in our lab and in late pre-clinical stage of development currently, possesses potent antibacterial and anti-MTB activity [9,11] as well as extremely low phototoxicity, hepatotoxicity and cardiac toxicity (unpublished data). However, IMB-070593 has low water solubility (0.4 mg/mL) and its mesylate salt still exhibits poor solubility (22.5 mg/mL). Since water solubility at physiological pH is important for preclinical testing, *in vivo* efficacy, and parenteral formulation [12], we decided to improve the solubility of IMB-070593 by employing a prodrug strategy.

One of the best strategies for increasing solubility of a drug containing a hydroxyl, carboxyl or an amino moiety is, in pharmaceutical field, to transform it into an amino acid prodrug, as exemplified by valaciclovir (a valine prodrug of acyclovir) [13]. We had previously reported that some of the amino acid prodrugs of tosufloxacin and moxifloxacin (MXFX) have better solubility and *in vivo* activity against clinically pathogens than the parent drugs [14,15].

Inspired by the above research results, it was decided to introduce common amino acids onto the nitrogen of the 3-amino-4-(methoxyimino)piperidine side chain of IMB-070593 by covalent binding. Thus, a series of amino acid and dipeptide prodrugs of IMB-070593 were designed, synthesized and evaluated for their water solubility and then antibacterial activity in this study. Our primary objective was to improve the solubility of IMB-070593, followed by optimizing its *in vivo* potency so as to finally develop the parenteral formulation.

## 2. Results and Discussion

### 2.1. Chemistry

**Scheme 1:**
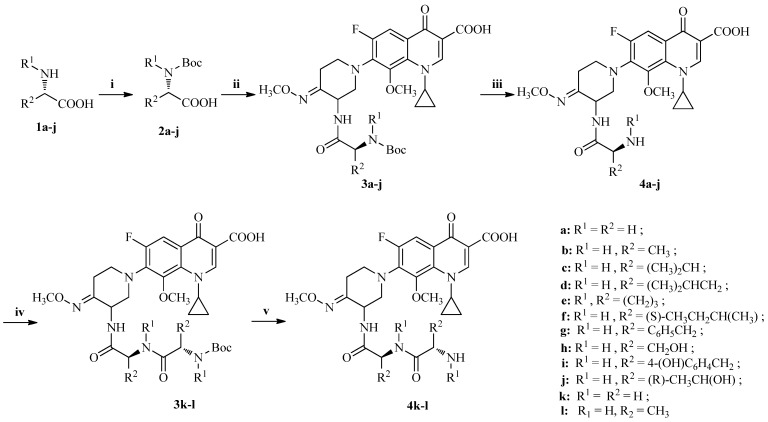
Synthesis of the prodrugs **4a**–**l** of IMB-070593.

Synthetic pathways to the prodrugs **4a**–**l** of IMB-070593 are depicted in Scheme 1. According to published procedures [14,15], treatment of various amino acids **1a**–**j** with di-*tert*-butyl dicarbonate (Boc_2_O) in methanol gave *N*-Boc-protected acids **2a**–**j**, which were coupled with IMB-070593 in the presence of dicyclohexylcarbodiimide (DCC) to yield the condensation products **3a**–**j**. The *N*-Boc protecting group on **3a**–**j** was removed with trifluoroacetic acid (TFA) to provide the amino acid prodrugs **4a**–**j**. Similarly, Gly-Gly-amino prodrug **4k** or L-Ala-L-Ala-amino prodrug **4l** could be conveniently obtained from the corresponding mono-Gly-amino prodrug **4a** and *N*-Boc-protected glycine **2a** or mono-L-Ala-amino prodrug **4b** and *N*-Boc-protected L-alanine **2b** according to the same procedures used for preparation of **4a**–**j**.

*Reagents and Conditions*: (i) (1) (Boc)_2_O, (C_2_H_5_)_3_N, CH_3_OH, (2) 2 N HCl; (ii) **IMB-070593**, DCC, CH_2_Cl_2_; (iii)/(v) (1) TFA, (2) NH_4_OH; (iv) **2a** or **2b**, DCC, CH_2_Cl_2_.

Because the methyloxime group of all the prodrugs **4a**–**l **may be present in the *E-* or *Z-* configuration, it was necessary to determine their geometries. Although we were unable to prepare X-ray quality single crystals of any methyloxime intermediate or product in this study, we had previously obtained the ingle crystals of 4-(methoxyimino)-3-methylaminopiperidine dihydrochloride, a *N*-methylated analogue of the side chain at C-7 position of IMB-070593, in which the piperidine ring adopts a chair conformation and the methyloxime geometry exists in an *E*-configuration [9]. Accordingly, we can speculate that the methyloxime group of the prodrugs in this study should have the same *E*-configuration.

### 2.2. Water Solubility

The prodrugs **4a**–**l** were initially evaluated for their water solubility which was determined by HPLC measurement of the concentration of a micromembrane filtered saturated solution [16]. The solubility of **4a**–**l** along with the parent IMB-070593 and IMB-070593 mesylate for comparison is presented in Table 1. The data reveal that all of **4a**–**l** have greater solubility (0.6–795.1 mg/mL) than that of IMB-070593 (0.4 mg/mL), and two dipeptide prodrugs **4k** and **4l** (235.0 and 281.5 mg/mL, respectively) are more soluble than the corresponding amino acid prodrugs **4a** and **4b** (97.0 and 112.0 mg/mL, respectively). It is encouraging that prodrugs **4a**,**b**,**e**,**f**,**k**,**l** have also much greater solubility (>85 mg/mL) than the parent IMB-070593 mesylate (22.5 mg/mL) which suggest that the six prodrugs could display better *in vivo* efficacy than IMB-070593 mesylate.

**Table 1:** Structures, physical data and solubility of compounds **4a**–**l**.

Compd.	R^1^	R^2^	m.p.[°C] ^a^	[α] 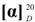 (c, CH_3_OH)	Water Solubility(mg/mL)
**4a**	H	H	136–137	0° (0.50)	112.0
**4b**	H	CH_3_	133–134	−2.80°(0.50)	97.0
**4c**	H	(CH_3_)_2_CH	154–156	−24.30°(0.50)	15.6
**4d**	H	(CH_3_)_2_CHCH_2_	109–110	−20.37°(0.11)	5.3
**4e**	(CH_2_)_3_		157–158	−19.17°(0.24)	795.1
**4f**	H	(S)-CH_3_CH_2_CH(CH_3_)	136–138	−26.68°(0.40)	87.4
**4g**	H	C_6_H_5_CH_2_	123–125	−13.32°(0.77)	1.0
**4h**	H	CH_2_OH	114–116	−9.01° (0.44)	50.2
**4i**	H	4-(OH)C_6_H_4_CH_2_	138–140	−14.14°(0.50)	0.6
**4j**	H	(R)-CH_3_CH(OH)	111–113	−15.25°(0.12)	72.3
**4k**	H	H	121–123	0° (0.13)	235.0
**4l**	H	CH_3_	120–121	−27.19°(0.23)	281.5
IMB					0.4
IMB mesylate					22.5

^a^ Melting points are uncorrected; IMB: IMB-070593.

### 2.3. Antibacterial Activity

#### 2.3.1. *In Vitro* Activity

The prodrugs **4a**–**l** were evaluated for their *in vitro* antibacterial activity against representative bacterial strains using standard techniques [17]. Minimum inhibitory concentration (MIC) is defined as the concentration of the compound required to give complete inhibition of bacterial growth, and MIC values of **4a**–**l** against Gram-positive and Gram-negative strains along with IMB-070593 mesylate, MXFX and levofloxacin (LVFX) for comparison, are listed in Table 2.

**Table 2:** *In vitro* activity of compounds **4a**–**l** against Gram-positive and Gram-negative strains.

Compd.	Strains MIC (μg/mL)														
	*S.a.*	MSSA	MRSA	MSSE	MRSE	*S.p.*	*E.coli*	*E.co.*1	*E.co.*2	*E.co.*3	*K.p.*1	*K.p.*2	*P.a.*1	*P.a.*2	*P.a.*3
**4a**	0.25	0.5	32	1	32	0. 5	4	16	128	>128	8	32	32	16	64
**4b**	0.125	0.25	64	1	32	1	4	16	128	>128	8	64	64	16	64
**4c**	0.25	0.25	64	2	64	1	4	16	>128	>128	16	32	32	32	64
**4d**	0.25	0.25	64	4	64	2	4	16	>128	>128	16	128	128	64	64
**4e**	0.25	2	64	4	64	2	4	32	>128	>128	16	64	64	128	128
**4f**	0.5	0.5	64	2	64	1	8	32	>128	>128	16	128	128	32	64
**4g**	0.125	0.25	64	1	64	1	16	64	>128	>128	64	>128	128	64	128
**4h**	1	1	64	2	64	1	16	128	>128	>128	32	>128	128	32	128
**4i**	2	2	>128	8	64	2	16	128	>128	>128	32	>128	128	32	128
**4j**	2	1	128	2	64	2	16	64	>128	>128	32	64	64	32	64
**4k**	4	8	>128	16	64	8	16	128	>128	>128	64	>128	>128	>128	128
**4l**	4	4	>128	32	64	8	32	>128	>128	>128	64	>128	128	>128	128
IMB mesylate	<0.008	<0.008	2	0.06	0.125	0.03	0.06	0.5	16	16	0.06	0.5	4	2	16
MXFX	<0.008	<0.008	8	0.125	0.25	0.06	<0.008	0.5	16	16	0.03	0.5	2	16	8
LVFX	0.06	0.06	64	0.5	0.03	0.125	<0.008	0.25	4	16	0.03	0.5	0.5	32	8

*S.a.*: *S. aureus* ATCC 25923. MSSA: Methicillin-sensitive *S. aureus* 12-4. MRSA: Methicillin-resistant *S. aureus* 12-1. MSSE: Methicillin-sensitive *S.epidermidis* 12-3. MRSE: Methicillin-resistant *S. epidermidis* 12-1. *S.p.*: *S. pneumoniae* ATCC 49619. *E.coli*: *E. coli* ATCC 25922. *E.co.*1: *E. coli* 12-6. *E.co.*2: *E. coli* 12-11. *E.co.*3: Extended-spectrum β-lactamase-producing (ESBL^+^) *E. coli* 12-14. *K.p.*1: *K. pneumoniae* 12-4. *K.p.*2: ESBL^+ ^*K. pneumoniae* 12-7. *P.a.1*: *P. aeruginosa* 12-12. *P.a.*2: *P. aeruginosa* 12-14. *P.a.*3: *P. aeruginosa* 12-20. IMB mesylate: IMB-070593 mesylate. MXFX: Moxifloxacin. LVFX: Levofloxacin.

The prodrugs **4a**–**l** show significantly less activity than the parent IMB-070593, MXFX and LVFX which is consistent with the biological character of common prodrugs. Moreover, **4a**–**l** are generally more active against Gram-positive strains except methicillin-resistant *S. aureus* (MRSA) and methicillin-resistant *S. epidermidis* (MRSE) than Gram-negative ones, and they have virtually no activity against all of the tested Gram-negative strains except for few exceptions.

#### 2.3.2. *In Vivo* Activity

Mice protection tests were used to evaluate *in vivo* efficacy of the prodrugs **4a**,**b**,**e**,**f**,**k**,**l** with greater solubility than IMB-070593 mesylate, given the fact that there is no obvious difference among of **4a**–**l** with regard to their *in vitro* activity. The efficacy of them was initially tested against two clinical isolate strains (methicillin-sensitive *S. aureus* / MSSA 12-1, *E. Coli* 12-1), and then **4a** of the amino acid prodrugs and **4k** of the dipeptide ones with better efficacy were chosen for further evaluation their protective effects *in vivo* (p.o.) against other four clinical isolates and that (i.v.) against the above two strains, IMB-070593 mesylate was used as the control drug (Table 3).

The results illustrate that all of the six prodrugs have better oral activity against MSSA 12-1 (6.44–9.39 mg/kg) than IMB-070593 mesylate (9.40 mg/kg), but weaker against *E. coli* 12-1*.* Furthermore, **4a** and **4k** show stronger efficacy than the parent drug against *S. pneumonia* 12-10 (p.o., 18.39 and 23.13 mg/kg, respectively) and MSSA 12-1 (i.v., 5.71 and 5.32 mg/kg, respectively), but weaker oral activity against MRSE 12-1, MRSA 12-5 and *K. pneumonia* 12-1. In a word, the prodrugs seem to be more active against drug-sensitive Gram-positive strains (p.o. or i.v., such as MSSA12-1 and *S. pneumonia* 12-10) than IMB-070593 mesylate. Conversely, they are much weaker against drug-resistant Gram-positive strains (p.o., such as MRSE 12-1and MRSA 12-5) and Gram-negative strains (p.o., such as *E. Coli* 12-1 and *K. pneumonia* 12-1), compared with the parent drug.

**Table 3:** *In vivo* efficacy of selected compounds against systemic infections in mice.

Infected strain(cfu/mL)	Compd	MIC(μg/mL)	ED_50_ (mg/kg)^a^ [95% confidence limit (mg/kg)]	
MSSA 12-1 (3.0 × 10^4^)	**4a**	1	7.16 (10.13–4.97) ^b^	5.71 (7.97–4.11) ^c^
	**4b**	0.5	9.30 (13.43–6.41)	NT ^d^
	**4e**	2	8.74 (12.17–6.27)	NT
	**4f**	1	6.44 (9.75–3.83)	NT
	**4k**	8	7.68 (10.76–5.42)	5.32 (7.58–3.74)
	**4l**	8	9.39 (13.85–6.31)	NT
	IMB mesylate	<0.008	9.40 (13.36–6.60)	7.15 (10.73–4.95)
*E. coli* 12-1 (6.0 × 10^5^)	**4a**	16	17.53 (25.25–12.04)	12.51 (19.41–8.37)
	**4b**	16	25.17 (37.89–17.12)	NT
	**4e**	32	21.44 (31.18–14.83)	NT
	**4f**	32	17.53 (25.25–12.04)	NT
	**4k**	128	18.80 (26.72–13.20)	14.30 (21.46–9.91)
	**4l**	>128	23.43 (36.23–15.57)	NT
	IMB mesylate	0.5	8.76 (12.63–6.02)	7.67 (11.83–5.27)
MRSE 12-1 (4.5 × 10^6^)	**4a**	32	50.55 (39.57–66.50)	NT
	**4k**	64	54.03 (40.89–77.35)	NT
	IMB mesylate	2	15.33 (11.47–20.06)	NT
MRSA 12-5 (4.5 × 10^6^)	**4a**	32	72.80 (55.03–123.15)	NT
	**4k**	>128	86.06 (67.10–152.78)	NT
	IMB mesylate	2	25.36 (20.10–32.68)	NT
*S.pneumonia*12-10 (5.2 × 10^8^)	**4a**	1	18.39 (14.00–23.60)	NT
	**4k**	2	23.13 (17.30–31.81)	NT
	IMB mesylate	0.06	27.57 (21.85–36.07)	NT
*K.pneumoniae* 12-1 (4.5 × 10^6^)	**4a**	4	33.91 (26.82–44.56)	NT
	**4k**	16	33.30(25.16–47.78)	NT
	IMB mesylate	0.06	18.56(14.55–23.97)	NT

^a^ ED_50_: 50% effective dose [95% confidence limit (mg/kg)]; ^b^ Antimicrobial agents were orally administrated twice at 0 h and 6 h after infection.; ^c^ Antimicrobial agents were intravenous injected once at 0 h after infection; ^d^ NT: not tested. MRSE: MRSE: Methicillin-resistant *S. epidermidis*. MRSA: Methicillin-resistant *S. aureus*.

## 3. Experimental

### 3.1. General Information

Melting points were determined in open capillaries and are uncorrected. ^1^H-NMR spectra were determined on a Varian Mercury-400/500/600 spectrometer in DMSO-*d_6_* or CDCl_3_ using tetramethylsilane (TMS) as an internal standard. Electrospray ionization (ESI) mass spectra and high resolution mass spectra (HRMS) were obtained on a MDSSCIEX Q-Tap mass spectrometer and AccuTOF CS JMS-T100CS (JEOL) mass spectrometer. Unless otherwise noted, the reagents were obtained from commercial supplier and used without further purification. TLC was performed on silica gel plates (Merck, ART5554 60F254).

### 3.2. Synthesis

#### 3.2.1. General Procedure for the Synthesis of **3a**–**j**

To a solution of amino acids **1a**–**j** (20 mmol) in methanol (90 mL) was added di-*tert*-butyl carbonate (8.73 g, 40 mmol) and triethylamine (11.1 mL, 80 mmol). The reaction mixture was heated to refluxing and stirred for 3 h at the same temperature, and concentrated under reduced pressure. The residue was diluted with water (40 mL), adjusted to pH 2.0–3.0 with 2 N HCl at 0–5 °C, and then extracted with ethyl acetate (50 mL × 3). The combined extracts were washed with saturated brine (30 mL), dried over anhydrous Na_2_SO_4_, and then concentrated under reduced pressure to provide **2a**–**j** as white solids [18,19,20,21,22,23] (64.8%–91.4%). A mixture of IMB-070593 (2.08 g, 4.97 mmol), **2a**–**j** (5.71 mmol), dicyclohexylcarbodiimide (1.18 g, 5.71 mmol) and dry dichloromethane (42 mL) was stirred at room temperature for 1 h and filtered. The filtrate was concentrated under reduced pressure, and the residue was treated with diethyl ether (20 mL), and then filtered. The solid was purified by column chromatography (silica gel) eluted with dichloromethane and methanol (v:v = 55:1) to afford the title compounds **3a**–**j** (36%–79%, from **1a**–**j**) as white or yellow solids.

*7-(3-(2-(tert-Butoxycarbonylamino)acetamido)-4-(methoxyimino)piperidin-1-yl)-1-cyclopropyl-6-fluoro-8-methoxy-4-oxo-1,4-dihydroquinoline-3-carboxylic acid* (**3a**). Obtained from **2a** and IMB-070593 as a white solid (86.1%), m.p.: 139–140 °C. ^1^H-NMR (400 MHz, CDCl_3_) δ (ppm) 14.68 (s, 1H, COOH), 8.83 (s, 1H, C_2_-H), 7.90 (d, *J* = 11.7 Hz, 1H, C_5_-H), 7.12 (d, *J* = 5.4 Hz, 1H), 5.09 (s, 1H), 4.70–4.59 (m, 1H), 4.18–4.14 (m, 1H), 4.04–4.01 (m, 1H), 3.92 (s, 3H, CH_3_O-N), 3.85 (d, *J* = 5.4 Hz, 1H), 3.79 (s, 3H, CH_3_O-C), 3.72 (q, *J* = 6.9 Hz, 1H), 3.62 (d, *J* = 9.0 Hz, 1H), 3.34 (d, *J* = 14.2 Hz, 1H), 3.26 (t, *J* = 11.9 Hz, 1H), 3.04 (t, *J* = 10.3 Hz, 1H), 2.34–2.32 (m, 1H), 1.47 (s, 9H, Boc), 1.30–1.19 (m, 2H, cyclopropyl CH_2_), 1.12–0.91 (m, 2H, cyclopropyl CH_2_). MS-ESI (*m/z*): 576.31(M+H)*^+^*.

*7-(3-((S)-2-(tert-Butoxycarbonylamino)propanamido)-4-(methoxyimino)piperidin-1-yl)-1-cyclopropyl-6-fluoro-8-methoxy-4-oxo-1,4-dihydroquinoline-3-carboxylic acid* (**3b**). Obtained from **2b** as a off-white solid (84.6%), m.p.: 150–152 °C. ^1^H-NMR (400 MHz, CDCl_3_) δ (ppm) 14.68 (s, 1H, COOH), 8.83 (s, 1H, C_2_-H), 7.91 (d, *J* = 11.6 Hz, 1H, C_5_-H), 7.27–7.07 (m, 1H), 4.98 (s, 1H), 4.63 (s, 1H), 4.21 (s, 1H), 4.22–4.10 (m, 1H), 4.03 (s, 1H), 3.92 (d, *J* = 2.6 Hz, 3H, CH_3_O-N), 3.81 (s, 3H, CH_3_O-C), 3.61 (d, *J* = 7.7 Hz, 1H), 3.36–3.24 (m, 2H), 3.03 (t, *J* = 10.8 Hz, 1H), 2.32 (s, 1H), 1.46 (d, *J* = 6.8 Hz, 9H, Boc), 1.38–1.26 (m, 3H, CH_3_), 1.25–1.13 (m, 2H, cyclopropyl CH_2_), 1.01 (s, 2H, cyclopropyl CH_2_). MS-ESI (*m/z*): 590.32(M+H)*^+^*.

*7-(3-((S)-2-(tert-Butoxycarbonylamino)-3-methylbutanamido)-4-(methoxyimino)piperidin-1-yl)-1-cyclopropyl-6-fluoro-8-methoxy-4-oxo-1,4-dihydroquinoline-3-carboxylic acid* (**3c**). Obtained from **2c** as a white solid (78.6%), m.p.: 130–131 °C. ^1^H-NMR (400 MHz, CDCl_3_) δ (ppm) 14.69 (s, 1H, COOH), 8.83 (s, 1H, C_2_-H), 7.91 (d, *J* = 11.6 Hz, 1H, C_5_-H), 7.01–6.89 (m, 1H), 5.06 (s, 1H), 4.73–4.58 (m, 1H), 4.19–4.08 (m, 1H), 4.07–4.01 (m, 1H), 3.92 (d, *J* = 8.3 Hz, 3H, CH_3_O-N), 3.80 (s, 3H, CH_3_O-C), 3.61 (s, 1H), 3.48 (q, *J* = 7.0 Hz, 1H), 3.39–3.20 (m, 2H), 3.06–2.99 (m, 1H), 2.31 (s, 1H), 2.21–2.08 (m, 1H, CH(CH_3_)_2_), 1.45 (d, *J* = 2.3 Hz, 9H, Boc), 1.23–1.21(m, 2H, cyclopropyl CH_2_), 1.09–0.88 (m, 8H, cyclopropyl CH_2_, CH(CH_3_)_2_). MS-ESI (*m/z*): 618.65 (M+H)*^+^*.

*7-(3-((S)-2-(tert-Butoxycarbonylamino)-4-methylpentanamido)-4-(methoxyimino)piperidin-1-yl)-1-cyclopropyl-6-fluoro-8-methoxy-4-oxo-1,4-dihydroquinoline-3-carboxylic acid* (**3d**). Obtained from **2d** as a yellow solid (58.2%), m.p.: 142–143 °C. ^1^H-NMR (500 MHz, CDCl_3_) δ (ppm) 14.67 (s, 1H, COOH), 8.83 (s, 1H, C_2_-H), 7.91 (d, *J* = 11.6 Hz, 1H, C_5_-H), 7.26–7.02 (m, 1H), 4.88 (d, *J* = 19.8 Hz, 1H), 4.64 (s, 1H), 4.14 (d, *J* = 17.1 Hz, 1H), 4.06–4.00 (m, 1H), 3.92 (d, *J* = 9.4 Hz, 3H, CH_3_O-N), 3.80 (d, *J* = 2.6 Hz, 3H, CH_3_O-C), 3.61 (s, 1H), 3.46 (d, *J* = 10.3 Hz, 1H), 3.39–3.21 (m, 2H), 3.03 (t, *J* = 10.5 Hz, 1H), 2.38–2.24 (m, 1H), 1.94 (d, *J* = 9.8 Hz, 1H), 1.70 (s, 2H), 1.45 (d, *J* = 6.4 Hz, 9H, Boc), 1.38–1.30 (m, 1H), 1.27 (d, *J* = 10.0 Hz, 1H), 1.21–1.13 (m, 2H, cyclopropyl CH_2_), 1.04–1.00 (m, 1H), 0.98–0.95 (m, 5H). MS-ESI (*m/z*): 632.63 (M+H)*^+^*.

*7-(3-((S)-1-(tert-Butoxycarbonyl)pyrrolidine-2-carboxamido)-4-(methoxyimino)piperidin-1-yl)-1-cyclopropyl-6-fluoro-8-methoxy-4-oxo-1,4-dihydroquinoline-3-carboxylic acid* (**3e**). The title compound **3e** was obtained from **2e** as a off-white solid (83.6%), m.p.: 186–187 °C. ^1^H-NMR (400 MHz, CDCl_3_) δ (ppm) 14.67 (s, 1H, COOH), 8.83 (s, 1H, C_2_-H), 7.91 (d, *J* = 11.5 Hz, 1H, C_5_-H), 7.00 (s, 1H), 4.63 (s, 1H), 4.22 (s, 1H), 4.10 (d, *J* = 15.4 Hz, 1H), 4.03 (d, *J* = 3.9 Hz, 1H), 3.91 (d, *J* = 2.6 Hz, 3H, CH_3_O-N), 3.81 (d, *J* = 4.8 Hz, 3H, CH_3_O-C), 3.70–3.17 (m, 6H), 3.03 (s, 1H), 2.47–2.10 (m, 2H), 2.00–1.84 (m, 2H), 1.46 (d, *J* = 10.9 Hz, 9H, Boc), 1.29–1.20 (m, 2H, cyclopropyl CH_2_), 1.00 (d, *J* = 4.4 Hz, 2H, cyclopropyl CH_2_). MS-ESI (m/z): 616.67 (M+H)*^+^*.

*7-(3-((2S,3S)-2-(tert-Butoxycarbonylamino)-3-methylpentanamido)-4-(methoxyimino)piperidin-1-yl)-1-cyclopropyl-6-fluoro-8-methoxy-4-oxo-1,4-dihydroquinoline-3-carboxylic acid* (**3f**). Obtained from **2f** as a white solid (96.5%), m.p. : 126–127 °C. ^1^H-NMR (400 MHz, CDCl_3_) δ (ppm) 14.68 (s, 1H, COOH), 8.83 (s, 1H, C_2_-H), 7.91 (d, *J* = 11.6 Hz, 1H, C_5_-H), 6.99 (d, *J* = 5.9 Hz, 1H), 5.08 (s, 1H), 4.67 (d, *J* = 4.1 Hz, 1H), 4.21–3.98 (m, 3H), 3.92 (d, *J* = 8.5 Hz, 3H, CH_3_O-N), 3.80 (s, 3H, CH_3_O-C), 3.61 (s, 1H), 3.35 (d, *J* = 14.1 Hz, 1H), 3.26 (s, 1H), 3.03 (d, *J* = 9.6 Hz, 1H), 2.30 (s, 1H), 1.90–1.82 (m, 2H), 1.46 (s, 9H, Boc), 1.29–1.16 (m, 3H), 1.01 (s, 2H), 0.95–0.91 (m, 6H). MS-ESI (*m/z*): 632.35 (M+H)*^+^*.

*7-(3-((S)-2-(tert-Butoxycarbonylamino)-3-phenylpropanamido)-4-(methoxyimino)piperidin-1-yl)-1-cyclopropyl-6-fluoro-8-methoxy-4-oxo-1,4-dihydroquinoline-3-carboxylic acid* (**3g**). Obtained from **2g** as a white solid (79.1%), m.p.: 149–150 °C. ^1^H-NMR (400 MHz, CDCl_3_) δ (ppm) 14.68 (s, 1H, COOH), 8.84 (s, 1H, C_2_-H), 7.92 (d, *J* = 11.6 Hz, 1H, C_5_-H), 7.40–7.22 (m, 5H, ph-H), 6.90–6.77 (m, 1H), 5.07–5.01 (m, 1H), 4.56 (s, 1H), 4.40 (s, 1H), 4.16–3.97 (m, 2H), 3.85 (d, *J* = 10.0 Hz, 3H, CH_3_O-N), 3.79 (s, 3H, CH_3_O-C), 3.56 (s, 1H), 3.31–2.75 (m, 5H), 2.28 (t, *J* = 12.4 Hz, 1H), 1.43 (s, 9H, Boc), 1.28–1.20 (m, 2H, cyclopropyl CH_2_), 1.03 (d, *J* = 11.3 Hz, 2H, cyclopropyl CH_2_). MS-ESI (*m/z*): 666.31 (M+H)*^+^*.

*7-(3-((S)-2-(tert-Butoxycarbonylamino)-3-hydroxypropanamido)-4-(methoxyimino)piperidin-1-yl)-1-cyclopropyl-6-fluoro-8-methoxy-4-oxo-1,4-dihydroquinoline-3-carboxylic acid* (**3h**). Obtained from **2h** as a off-white solid (83.3%), m.p. : 133–135 °C. ^1^H-NMR (400 MHz, CDCl_3_) δ (ppm) 14.64 (s, 1H, COOH), 8.83 (s, 1H, C_2_-H), 7.92 (d, *J* = 11.6 Hz, 1H, C_5_-H), 7.15 (s, 1H), 5.48 (s, 1H), 4.84–4.61 (m, 1H), 4.23 (s, 1H), 4.17–3.97 (m, 3H), 3.93 (d, *J* = 11.7 Hz, 3H, CH_3_O-N), 3.80 (d, *J* = 2.6 Hz, 3H, CH_3_O-C), 3.70–3.68 (m, 1H), 3.64–3.43 (m, 1H), 3.35–3.25 (m, 2H), 3.12 (t, *J* = 10.3 Hz, 1H), 2.37–2.31 (m, 1H), 1.91 (brs, 1H, OH), 1.47 (d, *J* = 10.2 Hz, 9H, Boc), 1.29–1.19 (m, 2H, cyclopropyl CH_2_), 1.03 (t, *J* = 7.7 Hz, 2H, cyclopropyl CH_2_). MS-ESI (*m/z*): 606.24 (M+H)*^+^*.

*7-(3-((S)-2-(tert-Butoxycarbonylamino)-3-(4-hydroxyphenyl)propanamido)-4-(methoxyimino)piperidin-1-yl)-1-cyclopropyl-6-fluoro-8-methoxy-4-oxo-1,4-dihydroquinoline-3-carboxylic acid* (**3i**). Obtained from **2i** as a off-white solid (43.9%), m.p.: 151–153 °C. ^1^H-NMR (400 MHz, CDCl_3_) δ (ppm) 14.73 (s, 1H, COOH), 8.82 (s, 1H, C_2_-H), 7.89–7.84 (m, 1H, C_5_-H), 7.02 (d, *J* = 8.1 Hz, 2H), 6.98–6.76 (m, 1H), 6.72 (d, *J* = 8.3 Hz, 2H), 5.14 (d, *J* = 35.9 Hz, 1H), 4.56 (s, 1H), 4.37 (s, 1H), 4.06 (d, *J* = 12.0 Hz, 2H), 3.87 (d, *J* = 10.9 Hz, 3H, CH_3_O-N), 3.80 (d, *J* = 4.7 Hz, 3H, CH_3_O-C), 3.76–3.46 (m, 2H), 3.22 (d, *J* = 11.9 Hz, 2H), 3.03–2.89 (m, 2H), 2.38–2.23 (m, 1H), 1.44 (s, 9H, Boc), 1.26–1.19 (m, 2H, cyclopropyl CH_2_), 1.12–0.94 (m, 2H, cyclopropyl CH_2_). MS-ESI (*m/z*): 682.22 (M+H)*^+^*.

*7-(3-((2S,3R)-2-(tert-Butoxycarbonylamino)-3-hydroxybutanamido)-4-(methoxyimino)piperidin-1-yl)-1-cyclopropyl-6-fluoro-8-methoxy-4-oxo-1,4-dihydroquinoline-3-carboxylic acid* (**3j**). Obtained from **2j** as a white solid (47.7%), m.p.: 134–136 °C. ^1^H-NMR (400 MHz, CDCl_3_) δ (ppm) 14.64 (s, 1H, COOH), 8.83 (s, 1H, C_2_-H), 7.92 (d, *J* = 11.6 Hz, 1H, C_5_-H), 7.33 (s, 1H), 5.42 (s, 1H), 4.74–4.65 (m, 1H), 4.42–4.33 (m, 1H), 4.17–4.03 (m, 3H), 3.92 (t, *J* = 4.3 Hz, 3H, CH_3_O-N), 3.80 (s, 3H, CH_3_O-C), 3.61 (s, 1H), 3.29 (dd, *J* = 22.8, 13.8 Hz, 2H), 3.11 (t, *J* = 11.4 Hz, 1H), 2.43–2.26 (m, 1H), 1.80 (brs, 1H, OH), 1.46 (d, *J* = 11.6 Hz, 9H, Boc), 1.26–1.20 (m, 5H, cyclopropyl CH_2_, CH_3_CH), 1.01 (s, 2H, cyclopropyl CH_2_). MS-ESI (*m/z*): 620.21 (M+H)*^+^*.

#### 3.2.2. General Procedure for the Synthesis of **4a**–**j**

To a stirring solution of trifluoroacetic acid (8.0 mL) was added **3a**–**j** (1.80 mmol) in portions over a period of 0.5 h at −5–0 °C and stirred for 3 h at the same temperature, and then concentrated under reduced pressure. The residue was treated with diethyl ether (10 mL) and then filtered. The solid was dissolved in methanol (2 mL) and adjusted to pH 7.0 with the ammonia water, and then extracted with dichloromethane (30 mL × 3). The combined extracts were washed with saturated brine (10 mL) and dried over anhydrous Na_2_SO_4_, and then concentrated under reduced pressure to provide the title compounds **4a**–**j**.

*7-(3-(2-Aminoacetamido)-4-(methoxyimino)piperidin-1-yl)-1-cyclopropyl-6-fluoro-8-methoxy-4-oxo-1,4-dihydroquinoline-3-carboxylic acid* (**4a**). Obtained from **3a** as a yellow solid (71.8%). ^1^H-NMR (400 MHz, CDCl_3_) δ (ppm) 8.83 (s, 1H, C_2_-H), 8.14 (s, 1H), 7.90 (d, *J* = 8.9 Hz, 1H, C_5_-H), 4.71 (s, 1H), 4.03–3.81 (m, 6H), 3.62 (s, 1H), 3.49–3.12 (m, 6H), 2.41 (s, 2H), 1.25–1.21 (m, 2H, cyclopropyl CH_2_), 1.02 (d, *J* = 17.5 Hz, 2H, cyclopropyl CH_2_). ^13^C-NMR (150 MHz, DMSO-d_6_) δ 176.33, 172.48, 165.59, 156.28, 154.37, 150.61, 146.23, 138.59(d, *J* = 12.2 Hz), 134.06, 121.49, 106.69, 106.46, 63.24, 61.33, 55.74, 49.15, 44.50, 40.73, 24.85, 18.55, 9.03, 8.86. MS-ESI (*m/z*): 476.23(M+H)^+^. HRMS-ESI (m/z): C_22_H_27_O_6_N_5_F, Calcd: 476.193899(M+H)^+^; Found: 476.19389(M+H)^+^.

*7-(3-((S)-2-Aminopropanamido)-4-(methoxyimino)piperidin-1-yl)-1-cyclopropyl-6-fluoro-8-methoxy-4-oxo-1,4-dihydroquinoline-3-carboxylic acid* (**4b**). Obtained from **3b** as a yellow solid (88.3%). ^1^H-NMR (400 MHz, CDCl_3_) δ (ppm) 8.81 (s, 1H, C_2_-H), 8.38–8.00 (m, 1H), 7.87 (d, *J* = 11.7 Hz, 1H, C_5_-H), 4.67 (s, 1H), 4.06 (s, 2H), 3.93 (s, 3H, CH_3_O-N), 3.82 (s, 3H, CH_3_O-C), 3.57 (d, *J* = 13.5 Hz, 2H), 3.31 (d, *J* = 11.1 Hz, 2H), 3.11 (s, 1H), 2.55–2.30 (m, 1H), 1.38–1.33 (m, 2H), 1.26–1.19 (m, 3H), 1.03 (s, 2H). ^13^C-NMR (100 MHz, CDCl_3_) δ 177.15, 175.65, 166.78, 157.70, 153.47, 150.12, 146.20, 139.02 (d, *J* = 12 Hz), 133.91, 122.77, 108.40, 107.96, 62.84, 62.06, 56.65, 51.00, 50.64, 50.24, 40.70, 25.67, 21.77, 9.86, 9.53. MS-ESI (*m/z*): 490.25 (M+H)^+^.HRMS-ESI (*m/z*): C_23_H_29_O_6_N_5_F, Calcd: 490.20964 (M+H)^+^; Found: 490.20968 (M+H)^+^.

*7-(3-((S)-2-Amino-3-methylbutanamido)-4-(methoxyimino)piperidin-1-yl)-1-cyclopropyl-6-fluoro-8-methoxy-4-oxo-1,4-dihydroquinoline-3-carboxylic acid* (**4c**). Obtained from **3c** as a light yellow solid (70.0%). ^1^H-NMR (400 MHz, DMSO-*d_6_*) δ (ppm) 8.70 (s, 1H, C_2_-H), 8.29–8.24 (m, 1H), 7.77 (d, *J* = 10.6 Hz, 1H, C_5_-H), 4.59–4.55 (m, 1H), 4.16 (s, 1H), 3.83 (s, 3H, CH_3_O-N), 3.76 (d, *J* = 5.2 Hz, 3H, CH_3_O-N), 3.73–3.58 (m, 1H), 3.56–3.41 (m, 1H), 3.28–3.16 (m, 1H), 3.03 (d, *J* = 3.5 Hz, 2H), 2.60–2.50 (m, 1H), 1.98–1.94 (m, 1H), 1.91–1.72 (m, 1H), 1.21–0.91 (m, 4H, 2 × cyclopropyl CH_2_), 0.87 (d, *J* = 6.8 Hz, 1H), 0.80 (d, *J* = 6.8 Hz, 2H), 0.74–0.70 (m, 3H). ^13^C-NMR (100 MHz, DMSO-*d_6_*) δ 176.30, 174.16, 165.61, 156.74, 154.45, 150.62, 146.08(d, *J* = 18 Hz), 138.60, 134.04, 121.33, 106.65, 106.42, 63.11, 61.27, 59.66, 55.47, 49.25, 40.71, 30.30, 24.76, 19.29, 16.65, 16.53, 8.98, 8.88. MS-ESI (*m/z*): 518.35 (M+H)^+^.HRMS-ESI (*m/z*): C_25_H_33_O_6_N_5_F, Calcd: 518.24094 (M+H)^+^; Found: 518.24080 (M+H)^+^.

*7-(3-((S)-2-Amino-4-methylpentanamido)-4-(methoxyimino)piperidin-1-yl)-1-cyclopropyl-6-fluoro-8-methoxy-4-oxo-1,4-dihydroquinoline-3-carboxylic acid* (**4d**). Obtained from **3d** as a yellow solid (71.5%). ^1^H-NMR (400 MHz, DMSO-d_6_) δ (ppm) 8.71 (s, 1H, C_2_-H), 8.46–8.23 (m, 1H), 7.77 (d, *J* = 10.6 Hz, 1H, C_5_-H), 4.55–4.49 (m, 1H), 4.17 (s, 1H), 3.83 (s, 3H, CH_3_O-N), 3.76 (d, *J* = 8.3 Hz, 3H, CH_3_O-C), 3.69 (s, 1H), 3.48–3.21 (m, 3H), 3.03 (t, *J* = 16.1 Hz, 1H), 2.64 (d, *J* = 6.4 Hz, 1H), 1.72 (s, 1H), 1.56 (s, 1H), 1.41 (s, 1H), 1.35–0.96 (m, 5H), 0.86–0.81 (m, 3H), 0.77–0.73 (m, 3H). ^13^C-NMR (100 MHz, DMSO-d_6_) δ 176.31, 175.04, 165.60, 156.72, 154.44, 150.60, 146.02 (d, *J* = 33 Hz), 138.66, 134.03, 121.43, 106.65, 106.42, 63.13, 61.27, 55.51, 52.99, 49.16, 44.07, 40.70, 24.87, 23.97, 23.12, 23.03, 21.85, 8.97, 8.93. MS-ESI (*m/z*): 532.40 (M+H)^+^. HRMS-ESI (*m/z*): C_26_H_35_O_6_N_5_F, Calcd: 532.25659 (M+H)^+^; Found: 532.25671 (M+H)^+^.

*7-(4-(methoxyimino)-3-((S)-pyrrolidine-2-carboxamido)piperidin-1-yl)-1-Cyclopropyl-6-fluoro-8-methoxy-4-oxo-1,4-dihydroquinoline-3-carboxylic acid* (**4e**). Obtained from **3e** as a yellow solid (66.8%). ^1^H-NMR (400 MHz, CDCl_3_) δ (ppm) 8.81 (s, 1H, C_2_-H), 8.45–8.39 (m, 1H), 7.87 (d, *J* = 11.7 Hz, 1H, C_5_-H), 4.67 (s, 1H), 4.04–3.98 (m, 2H), 3.92 (d, **J** = 7.8 Hz, 3H, CH_3_O-N), 3.81 (s, 4H), 3.57 (d, *J* = 12.5 Hz, 1H), 3.31–3.27 (m, 2H), 3.15 (t, *J* = 10.7 Hz, 1H), 3.05–2.93 (m, 2H), 2.51–2.31 (m, 1H), 2.15–2.10 (m, 1H), 2.00–1.95 (m, 1H), 1.88–1.56 (m, 2H), 1.24–1.19 (m, 2H, cyclopropyl CH_2_), 1.02 (s, 2H). ^13^C-NMR (100 MHz, CDCl_3_) δ 177.12, 175.17, 166.76, 157.61, 153.61, 150.11, 146.10, 138.97 (d, *J* = 12 Hz), 133.91, 122.64, 108.41, 107.97, 62.81, 61.96, 60.82, 56.59, 50.52, 49.88, 47.37, 40.68, 30.92, 26.21, 25.61, 9.86, 9.53. MS-ESI (*m/z*): 516.36 (M+H)^+^.HRMS-ESI (*m/z*): C_25_H_31_O_6_N_5_F, Calcd: 516.22529 (M+H)^+^; Found: 516.22545 (M+H)^+^.

*7-(3-((2S,3S)-2-Amino-3-methylpentanamido)-4-(methoxyimino)piperidin-1-yl)-1-cyclopropyl-6-fluoro-8-methoxy-4-oxo-1,4-dihydroquinoline-3-carboxylic acid* (**4f**). Obtained from **3f** as a yellow solid (88.3%). ^1^H-NMR (400 MHz, CDCl_3_) δ (ppm) 8.73 (d, *J* = 3.5 Hz, 1H, C_2_-H), 7.91–7.57 (m, 1H, C_5_-H), 7.46 (s, 1H), 4.71 (s, 1H), 4.13–4.04 (m, 3H), 3.90 (d, *J* = 6.9 Hz, 3H, CH_3_O-N), 3.79 (s, 3H, CH_3_O-C), 3.57–3.47 (m, 1H), 3.34–3.19 (m, 2H), 2.41 (s, 1H), 2.13 (s, 1H), 1.63–1.58 (m, 1H), 1.22 (d, *J* = 6.4 Hz, 4H), 1.06–0.97 (m, 6H), 0.92 (s, 2H). ^13^C-NMR (100 MHz, CDCl_3_) δ 176.74, 175.34, 166.81, 154.83, 153.05, 150.25, 145.93(d, *J* = 12 Hz), 138.91, 134.05, 122.08, 107.94, 107.61, 63.12, 62.15, 58.19, 55.96, 50.94, 50.48, 40.87, 37.01, 25.38, 14.86, 11.75, 9.76. MS-ESI (*m/z*): 532.33 (M+H)^+^.HRMS-ESI (*m/z*): C_26_H_35_O_6_N_5_F, Calcd: 532.25659 (M+H)^+^; Found: 532.25653 (M+H)^+^.

*7-(3-((S)-2-Amino-3-phenylpropanamido)-4-(methoxyimino)piperidin-1-yl)-1-cyclopropyl-6-fluoro-8-methoxy-4-oxo-1,4-dihydroquinoline-3-carboxylic acid* (**4g**). Obtained from **3g** as a yellow solid (82.4%). ^1^H-NMR (400 MHz, DMSO-*d_6_*) δ (ppm) 8.72 (s, 1H, C_2_-H), 8.28 (d, *J* = 4.6 Hz, 1H), 7.90–7.70 (m, 1H, C_5_-H), 7.40–6.90 (m, 5H, ph-H), 4.54 (s, 1H), 4.17 (s, 1H), 3.83 (d, *J* = 8.0 Hz, 3H, CH_3_O-N), 3.76–3.66 (m, 4H), 3.47 (d, *J* = 5.7 Hz, 2H), 3.31 (s, 2H), 3.23–3.07 (m, 1H), 2.96–2.91 (m, 2H), 2.74–2.51 (m, 1H), 1.26–0.83 (m, 4H, 2 × cyclopropyl CH_2_). ^13^C-NMR (100 MHz, DMSO-*d_6_*) δ 176.29, 173.76, 165.66, 156.71, 154.33, 150.64, 146.24, 138.35(d, *J* = 29 Hz), 134.00, 129.24, 128.02, 126.01, 121.57, 106.69, 106.46, 63.14, 61.30, 56.00, 55.68, 49.25, 40.68, 40.45, 24.70, 16.37, 8.95. MS-ESI (*m/z*): 566.40 (M+H)^+^. HRMS-ESI (*m/z*): C_29_H_33_O_6_N_5_F, Calcd: 566.24094 (M+H)^+^; Found: 566.24108 (M+H)^+^.

*7-(3-((S)-2-Amino-3-hydroxypropanamido)-4-(methoxyimino)piperidin-1-yl)-1-cyclopropyl-6-fluoro-8-methoxy-4-oxo-1,4-dihydroquinoline-3-carboxylic acid* (**4h**). Obtained from **3h** as a yellow solid (66.2%). ^1^H-NMR (400 MHz, DMSO-*d_6_*) δ (ppm) 8.71 (s, 1H, C_2_-H), 8.36 (d, *J* = 5.1 Hz, 1H), 7.78 (d, *J* = 8.0 Hz, 1H, C_5_-H), 4.76 (s, 1H), 4.56–4.52 (m, 1H), 4.17 (s, 1H), 3.84 (d, *J* = 0.9 Hz, 3H, CH_3_O-N), 3.81–3.70 (m, 4H), 3.60–3.35 (m, 4H), 3.26–3.25 (m, 2H), 3.11–2.96 (m, 1H), 2.56–2.51 (m, 1H), 1.13–0.96 (m, 4H, 2 × cyclopropyl CH_2_). ^13^C-NMR (100 MHz, DMSO-*d_6_*) δ 176.36, 172.77, 165.60, 156.82, 154.37, 150.61, 146.29 (d, *J* = 11 Hz), 138.67, 134.05, 121.49, 106.69, 106.42, 64.90, 63.18, 61.34, 56.76, 55.65, 49.40, 40.75, 24.85, 15.16, 8.92. MS-ESI (*m/z*): 506.28 (M+H)^+^.HRMS-ESI (*m/z*): C_23_H_29_O_7_N_5_F, Calcd: 506.20455 (M+H)^+^; Found: 506.20465 (M+H)^+^.

*7-(3-((S)-2-Amino-3-(4-hydroxyphenyl)propanamido)-4-(methoxyimino)piperidin-1-yl)-1-cyclopropyl-6-fluoro-8-methoxy-4-oxo-1,4-dihydroquinoline-3-carboxylic acid* (**4i**). Obtained from **3i** as a light yellow solid (83.4%). ^1^H-NMR (400 MHz, DMSO-*d_6_*) δ (ppm) 9.11 (d, *J* = 15.9 Hz, 1H), 8.71 (d, *J* = 4.5 Hz, 1H, C_2_-H), 8.23 (t, *J* = 8.0 Hz, 1H), 7.79 (dd, *J* = 11.8, 6.1 Hz, 1H, C_5_-H), 6.95 (t, *J* = 7.6 Hz, 2H), 6.59 (dd, *J* = 14.6, 8.3 Hz, 2H), 4.53 (s, 1H), 4.18 (dd, *J* = 6.8, 3.8 Hz, 1H), 3.83 (d, *J* = 7.8 Hz, 3H, CH_3_O-N), 3.74 (d, *J* = 10.8 Hz, 3H, CH_3_O-C), 3.67 (d, *J* = 7.8 Hz, 1H), 3.54–3.44 (m, 1H), 3.42–3.34 (m, 2H), 3.26–3.16 (m, 1H), 3.15–2.89 (m, 2H), 2.79 (d, *J* = 11.0 Hz, 1H), 2.65–2.53 (m, 1H), 1.16–1.01 (m, 4H, 2 × cyclopropyl CH_2_). ^13^C-NMR (100 MHz, DMSO-*d_6_*) δ 176.40, 173.94, 165.64, 156.74, 155.78, 154.30, 150.63, 146.26, 138.72(d, *J* = 38 Hz), 134.07, 130.27, 130.09, 128.33, 127.95, 121.53, 114.88, 106.69, 106.48, 63.15, 61.31, 56.18, 55.80, 49.71, 49.24, 40.75, 24.87, 15.15, 9.06, 8.96. MS-ESI (*m/z*): 582.30 (M+H)^+^.HRMS-ESI (*m/z*): C_29_H_33_O_7_N_5_F, Calcd: 582.23585 (M+H)^+^; Found: 582.23593 (M+H)^+^.

*7-(3-((2S,3R)-2-Amino-3-hydroxybutanamido)-4-(methoxyimino)piperidin-1-yl)-1-cyclopropyl-6-fluoro-8-methoxy-4-oxo-1,4-dihydroquinoline-3-carboxylic acid* (**4*J***). Obtained from **3*J*** as a yellow solid (73.3%). ^1^H-NMR (400 MHz, DMSO-*d_6_*) δ (ppm) 8.71 (s, 1H, C_2_-H), 8.38 (d, *J* = 7.0 Hz, 1H), 7.78 (dd, *J* = 11.8, 3.1 Hz, 1H, C_5_-H), 4.63–4.50 (m, 1H), 4.22–4.10 (m, 1H), 3.91–3.80 (m, 4H), 3.79–3.59 (m, 4H), 3.51–3.47 (m, 1H), 3.31–3.12 (m, 2H), 3.10–3.02 (m, 1H), 2.99 (d, *J* = 4.1 Hz, 1H), 2.60–2.51 (m, 1H), 1.29–1.23 (m, 1H), 1.13–1.11 (m, 2H), 1.04–0.97 (m, 3H), 0.91–0.73 (m, 1H). ^13^C-NMR (100 MHz, DMSO-*d6*) δ 176.34, 173.04, 165.60, 156.84, 154.39, 150.61, 146.10, 138.56 (d, *J* = 38 Hz), 134.06, 122.24, 106.66, 106.42, 67.14, 63.17, 61.32, 60.19, 55.60, 49.47, 39.94, 24.85, 20.08, 19.94, 8.94. MS-ESI (*m/z*): 520.28 (M+H)^+^. HRMS-ESI (*m/z*): C_24_H_31_O_7_N_5_F, Calcd: 520.22020 (M+H)^+^; Found: 520.22083 (M+H)^+^.

#### 3.2.3. General Procedure for the Synthesis of **3k**–**l**

A mixture of **4a**–**b** (0.82 mmol), **2a**–**b** (0.82 mmol), dicyclohexylcarbodiimide (0.17 g, 0.82 mmol) and dry dichloromethane (20 mL) was stirred at room temperature for 4 h, filtered and concentrated under reduced pressure. The solid was treated with diethyl ether (3 mL) and filtered. The solid was purified by column chromatography (silica gel) eluted with dichloromethane and methanol (v:v = 60:1) to afford the title compounds **3k**–**l**.

*7-(3-(2-(2-(tert-Butoxycarbonylamino)acetamido)acetamido)-4-(methoxyimino)piperidin-1-yl)-1-cyclopropyl-6-fluoro-8-methoxy-4-oxo-1,4-dihydroquinoline-3-carboxylic acid* (**3k**). Obtained from **4a** and **2a** as a light yellow solid (30.8%), m.p.: 122–124 °C.^1^H-NMR (400 MHz, CDCl_3_) δ (ppm) 14.71 (s, 1H, COOH), 8.83 (s, 1H, C_2_-H), 7.90 (d, *J* = 11.6 Hz, 1H, C_5_-H), 6.97 (d, *J* = 5.6 Hz, 1H), 6.75 (s, 1H), 5.07 (s, 1H), 4.72–4.56 (m, 1H), 4.11 (d, *J* = 7.0 Hz, 1H), 4.06–3.95 (m, 3H), 3.93 (s, 3H, CH_3_O-N), 3.86 (d, *J* = 3.7 Hz, 2H), 3.78 (s, 3H, CH_3_O-C), 3.61 (d, *J* = 10.0 Hz, 1H), 3.34–3.24 (m, 2H), 3.07 (t, *J* = 10.5 Hz, 1H), 2.42–2.26 (m, 1H), 1.45 (s, 9H, Boc), 1.26–1.21 (m, 2H, cyclopropyl CH_2_), 1.07–0.98 (m, 2H, cyclopropyl CH_2_). MS-ESI (*m/z*): 633.17 (M+H)*^+^*.

*7-(3-((S)-2-((S)-2-(tert-Butoxycarbonylamino)propanamido)propanamido)-4-(methoxyimino)piperidin-1-yl)-1-cyclopropyl-6-fluoro-8-methoxy-4-oxo-1,4-dihydroquinoline-3-carboxylic acid* (**3l**). Obtained from **4b** and **2b** as a yellow solid (71.5%), m.p.: 126–128 °C. ^1^H-NMR (400 MHz, CDCl_3_) δ (ppm) 14.68 (s, 1H, COOH), 8.83 (s, 1H, C_2_-H), 7.90 (d, *J* = 11.6 Hz, 1H, C_5_-H), 7.18–6.92 (m, 1H), 6.71 (d, *J* = 20.8 Hz, 1H), 4.93 (s, 1H), 4.70–4.58 (m, 1H), 4.53–4.48 (m, 1H), 4.16 (s, 1H), 4.16–4.03 (m, 2H), 3.92 (d, *J* = 4.0 Hz, 3H, CH_3_O-N), 3.79 (d, *J* = 1.5 Hz, 3H, CH_3_O-C), 3.59 (s, 1H), 3.40–3.18 (m, 2H), 3.14–2.97 (m, 1H), 2.34–2.32 (m, 1H), 1.63–1.29 (m, 15H, Boc, 2 × CH_3_), 1.28–1.16 (m, 2H, cyclopropyl CH_2_), 1.09–0.87 (m, 2H, cyclopropyl CH_2_). MS-ESI (*m/z*): 661.33 (M+H)*^+^*.

#### 3.2.4. General Procedure for the Synthesis of **4k**–**l**

To a stirring solution of trifluoroacetic acid (3.0 mL) was added **3k**–**l** (0.47 mmol) in portions over a period of 0.5 h at −5–0 °C, stirred for 2 h at the same temperature and then concentrated under reduced pressure. The residue was treated with diethyl ether (7 mL) and filtered. The solid was dissolved in methanol (1 mL) and adjusted to pH 7.0 with the ammonia water, and then extracted with dichloromethane (50 mL × 3). The combined extracts were washed with saturated brine (10 mL), dried over anhydrous Na_2_SO_4_, and concentrated under reduced pressure to provide the title compounds **4k**–**l**.

*7-(3-(2-(2-Aminoacetamido)acetamido)-4-(methoxyimino)piperidin-1-yl)-1-cyclopropyl-6-fluoro-8-methoxy-4-oxo-1,4-dihydroquinoline-3-carboxylic acid* (**4k**). Obtained from **3k** as a yellow solid (73.3%). ^1^H-NMR (400 MHz, CDCl_3_) δ (ppm) 8.81 (s, 1H, C_2_-H), 7.98–7.80 (m, 2H), 7.11 (d, *J* = 5.9 Hz, 1H), 4.75–4.56 (m, 1H), 4.14–4.10 (m, 1H), 4.07–4.00 (m, 2H), 3.97 (d, *J* = 5.6 Hz, 1H), 3.92 (s, 3H, CH_3_O-N), 3.79 (s, 3H, CH_3_O-C), 3.60 (d, *J* = 8.0 Hz, 1H), 3.52–3.44 (m, 1H), 3.44 (s, 1H), 3.35–3.19 (m, 2H), 3.09 (t, *J* = 10.7 Hz, 1H), 2.46–2.22 (m, 1H), 1.27–1.18 (m, 2H, cyclopropyl CH_2_), 1.13–0.95 (m, 2H, cyclopropyl CH_2_). ^13^C-NMR (100 MHz, CDCl_3_) δ 177.07, 173.52, 168.75, 166.74, 157.52, 152.99, 150.12, 146.05, 138.85(d, *J* = 12 Hz), 133.94, 122.74, 108.33, 107.96, 62.95, 62.11, 56.43, 50.57, 50.15, 44.79, 42.87, 40.70, 25.66, 9.86, 9.53. MS-ESI (*m/z*): 533.32 (M+H)^+^.HRMS-ESI (*m/z*): C_24_H_30_O_7_N_6_F, Calcd: 533.21545 (M+H)^+^; Found: 533.21660 (M+H)^+^.

*7-(3-((S)-2-((S)-2-Aminopropanamido)propanamido)-4-(methoxyimino)piperidin-1-yl)-1-cyclopropyl-6-fluoro-8-methoxy-4-oxo-1,4-dihydroquinoline-3-carboxylic acid* (**4l**). Obtained from **3l** as a yellow solid (34.7%). ^1^H-NMR (400 MHz, CDCl_3_) δ (ppm) 8.81 (s, 1H, C_2_-H), 7.86 (dd, *J* = 11.6, 6.2 Hz, 1H, C_5_-H), 7.75 (t, *J* = 7.8 Hz, 1H), 7.28–7.10 (m, 1H), 4.66–4.62 (m, 1H), 4.56–4.47 (m, 1H), 4.13–4.00 (m, 2H), 3.92 (d, *J* = 4.6 Hz, 3H, CH_3_O-N), 3.80 (s, 3H, CH_3_O-C), 3.68–3.44 (m, 2H), 3.32–3.25 (m, 2H), 3.08 (t, *J* = 10.4 Hz, 1H), 2.34 (t, *J* = 12.7 Hz, 1H), 1.44–1.33 (m, 5H), 1.26–1.21 (m, 3H), 1.03 (s, 2H). ^13^C-NMR (100 MHz, CDCl_3_) δ 177.11, 175.83, 172.12, 166.76, 159.46, 153.12, 150.11, 146.14(d, *J* = 12 Hz), 138.80, 133.90, 122.82, 108.39, 107.98, 62.90, 62.07, 56.37, 50.78, 50.60, 48.68, 48.47, 40.69, 25.69, 21.66, 18.13, 9.80, 9.58. MS-ESI (*m/z*): 561.44 (M+H)^+^.HRMS-ESI (*m/z*): C_26_H_34_O_7_N_6_F, Calcd: 561.24675 (M+H)^+^; Found: 561.24691 (M+H)^+^.

### 3.3. MIC Determination

All compounds were screened for their *in vitro* antibacterial activity against representative Gram-positive and Gram-negative strains, by means of standard twofold serial dilution method using agar media [17]. Minimum inhibitory concentration (MIC) is defined as the minimum concentration of the compound required to give complete inhibition of bacterial growth after incubation at 35 °C for 18–24 h.

## 4. Conclusions

In summary, a series of amino acid and dipeptide prodrugs of IMB-070593 were designed, synthesized and evaluated for their water solubility and antibacterial activity in this study. Our results reveal that the solubility (>85 mg/mL) of four amino acid prodrugs **4a**,**b**,**e**,**f** and two dipeptide prodrugs **4k**,**l** was much greater than that of IMB-070593 mesylate (22.5 mg/mL). Compounds **4a** and **4k** were found to have lower ED_50_ values when administered by intravenous injection, compared with orally. Moreover, both of them show stronger efficacy against drug-sensitive Gram-positive strains than the parent drug, as opposed to Gram-negative and drug-resistant Gram-positive strains.
